# Welding complex-shaped actuators from dynamic liquid crystal elastomers†

**DOI:** 10.1039/d5tc01194a

**Published:** 2025-05-21

**Authors:** Jie Jiang, Hongshuang Guo, Hao Zeng, Arri Priimagi

**Affiliations:** a Smart Photonic Materials, Faculty of Engineering and Natural Sciences, Tampere University P.O. Box 541 Tampere FI-33101 Finland Hongshuang.Guo@tuni.fi Arri.Priimagi@tuni.fi

## Abstract

Liquid crystal elastomers (LCEs) are promising materials for constructing and programming soft actuators and small-scale soft robotic systems due to their exceptional stimuli-responsive properties. However, fabricating complex-shaped LCE actuators with controlled shape transformations remains challenging. Herein, we present a welding-based strategy for fabricating light-responsive, multicomponent LCEs with complex shape morphing capabilities while preserving the distinct functional responses of individual components. This approach leverages dynamic disulfide bonds incorporated into surface-aligned, chain-extended LCEs, which enables robust adhesion between LCE segments without disrupting their molecular orientation during welding. The resulting structures seamlessly integrate differently oriented LCE segments, enabling diverse shape-morphing and establishing a platform for weldable, arbitrarily aligned, and complex-shaped LCE actuators.

## Abstract 2

A weldable liquid crystal elastomer (LCE) with dynamic disulfide bonds enables complex shape transformations *via* cut-and-paste assembly of surface-aligned LCE segments while preserving mesogen alignment.

## Graphical abstract

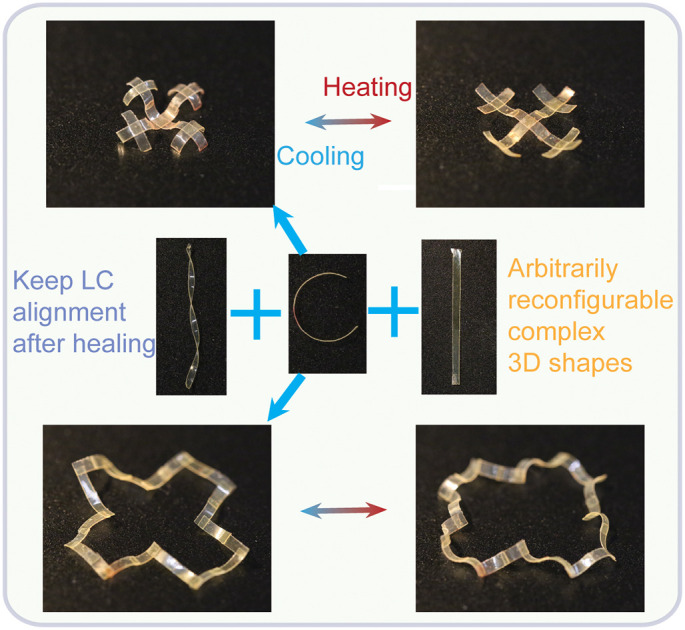

## Introduction

Stimuli-responsive liquid crystal networks (LCNs) and elastomers (LCEs; loosely crosslinked LCNs) are among the most promising material candidates for micro-actuators,^1–5^ artificial muscles,^6–8^ and miniature soft robotics.^9–12^ LCEs possess both mesogenic anisotropy and polymer elasticity, and this combination enables fast and reversible stimuli-induced deformation with programmable actuation modes.^13–16^ Precise alignment control of the liquid crystal molecules within the network is the key to versatile deformation modes from a fabricated strip or film.^17,18^ The deformation is driven by stimuli-induced disruption of the molecular alignment, yielding contraction along the director orientation and expansion perpendicular to it.^19,20^ Traditional photopolymerization-based fabrication methods^21^ can easily yield planar, twisted or splay-aligned LCEs, which can be spatially patterned to produce more complex actuation behavior.^22–25^ While 4D printing enables the creation of upscaled and complex shapes,^26–30^ it remains limited in its ability to precisely control director alignment, which is predominantly dictated by shear forces during the printing process.

One potential pathway towards complex 3D deformations is integrating several pre-fabricated LCE components with different molecular orientation patterns, and combining them synergistically to obtain a desired actuation mode.^31,32^ This can be achieved through physical adhesion, such as tape or glue.^12,33–35^ The disadvantage of this method is that glue at the bonding interface introduces defects, which can reduce compatibility of different parts and shorten the service life of the actuators. These drawbacks can be addressed *via* chemical methods, for which there are currently two main approaches. The first approach leans on a monolithic integration of multiple LCE films or strips with an excess of photopolymerizable groups into a single actuating structure, enabling multimaterial robotic constructs or laminated LCEs with increased work capacity.^31,36^ The second approach is based on dynamic bonds such as transesterification,^37,38^ disulfide or diselenide metathesis,^39–44^ siloxane exchange,^45,46^ halogen bonding,^47^ Diels–Alder reaction,^48,49^ or hydrogen bonding^50^ to obtain mechanical re-shaping and cut-and-paste welding or self-healing in LCE actuators. To ensure deformability, the dynamic-bond-based methods typically require a mechanical treatment step^38,39,42,43,45,48,51,52^ − stretching a partially cross-linked LCE and then locking the molecular orientation upon crosslinking – to obtain the desired molecular alignment and actuation behavior. The reorganization of the dynamic bonds can disrupt the liquid crystal alignment. Thus, re-stretching is often required for the material to recover responsiveness after the welding/healing process, essentially limiting the method to uniaxial LC orientation and shrinkage-based deformation. As exceptions to this, Hebner *et al.* have reported surface-enforced alignment of dynamic LCEs bearing allyl dithiols for complex, reprogrammable shape morphing,^53^ and Chen *et al.* used surface-aligned LCEs comprising side-on mesogens and diacrylate disulfide crosslinkers for cut-and-paste fabrication of soft actuators.^54^

In this work, we introduce disulfide bonds into surface-aligned chain-extended LCEs obtained *via* aza–Michael addition,^55,56^ and develop weldable LCE constructs with desired molecular alignment within each piece. Importantly, disulfide metathesis occurs under only moderate heating without catalysts or initiators. As a result, disulfide bonds are highly reversible and exhibit greater strength than most other dynamic bonds. We demonstrate that their inherent light absorption enables photothermal functionality without requiring the incorporation of external dyes. The proposed method offers deformation diversity upon heat or light stimulation with no need for additional mechanical alignment steps, enabling the construction of complex actuating shapes by cutting and pasting differently aligned LCE strips. We demonstrate the importance of the disulfide bonds in offering both the dynamism required for cut-and-paste welding and acting as near-UV absorber that enables photothermal actuation. The dynamics of disulfide bonds, combined with chain extension methodologies, provide a versatile platform for designing and fabricating weldable LCEs, expanding their potential as complex-shaped soft actuators.

## Results and discussion

### Sample fabrication

**Fig. 1:**
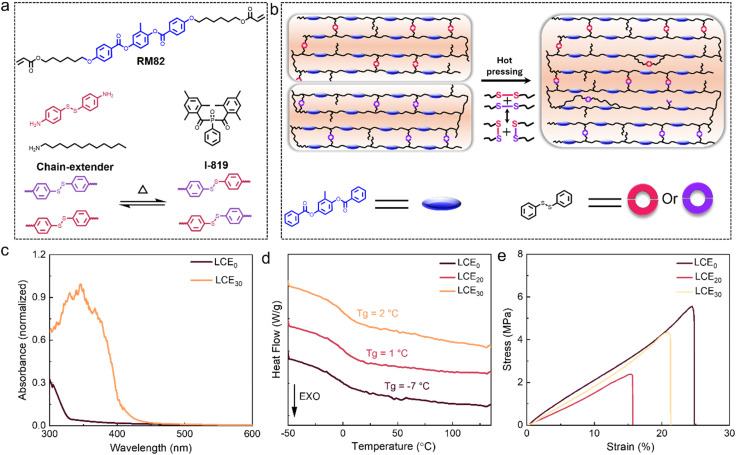
Disulfide-containning, chain-extended LCEs. (a) Chemical composition of the liquid crystal monomer mixture and schematic illustration of the disulfide bond metathesis. (b) Schematic illustration of the dynamic bond exchange due to disulfide metathesis. (c) Normalized absorption spectra of planar-aligned LCE_0_ and LCE_30_ films (thickness 5 μm) (d) DSC curves of LCE_0_, LCE_20_, and LCE_30_ during the second cooling cycle (10 °C min^−1^). (e) Tensile stress–strain curves of splay-aligned LCE_0_, LCE_20_, and LCE_30_ at room temperature.

Our materials design is based on the two-step fabrication method introduced by Ware *et al.*^52^ where the monomer mixture is first oligomerized in a surface-aligned cell, and then the molecular alignment is locked by photopolymerization (Fig. S1a, ESI†). Unless otherwise stated, the chemical composition consists of the widely used difunctional mesogen RM82,^21^ the chain extender dodecanamine, 4,4′-dithiodianiline, and the photoinitiator I-819 (Fig. 1a). Dodecanamine serves to adjust the mechanical properties of the LCE by reducing the crosslink density, while 4,4′-dithiodianiline is expected to serve as both chain extender and crosslinker.^57^ The dynamic disulfide bonds also endow the polymerized network with welding/self-healing properties.^39–42^ A surface anchoring method, *i.e.* rubbed cell preparation, is applied to align the LC molecules during the first reaction step (oligomerization), while the LC alignment is permanently fixed during the second reaction step (photopolymerization). Fig. 1b schematically illustrates the welding mechanism in the disulfide-containing chain-extended LCEs, through dynamic bond exchange during thermo-activated disulfide metathesis.^58^ In some experiments, 4,4′-dithiodianiline was replaced with cystamine or 4,4′-diaminodiphenylmethane (Fig. S1a, ESI†), to further elaborate the effect of the disulfide bonds on the properties of the LCEs.

LCEs with varying 4,4′-dithiodianiline content with respect to RM82, ranging from 0 mol% (LCE_0_) to 30 mol% (LCE_30_), were investigated, whereas the molar ratio between the diacrylate and amino functionalities was fixed to 1 : 0.8 in all samples (Fig. S1b, ESI†). If the 4,4′-dithiodianiline was further increased to 40 mol%, the sample crystallized and did not exhibit LC behavior. The FTIR spectra of LCE_20_ and LCE_30_ display a new band that can be associated with –NH group at 3400 cm^−1^ compared with LCE_0_, indicating successful introduction of 4,4′-dithiodianiline into the polymer networks.^57^ The UV-Vis absorption spectra of LCE_0_ and LCE_30_ (Fig. 1c) show that the introduction of 4,4′-dithiodianiline gives rise to an absorption band with a maximum around 350 nm.^59^ This enables photothermal control over the LCE shape changes, as will be demonstrated later.

Differential scanning calorimetry (DSC) indicates a modest increase of the glass transition temperature (*T*_g_) upon addition of 4,4′-dithiodianiline: −7 °C for LCE_0_, 1 °C for LCE_20_, and 2 °C for LCE_30_ (Fig. 1d and Fig. S3, ESI†). The mechanical properties of splay-aligned LCE_0_, LCE_20_, and LCE_30_ are shown in Fig. 1e. The elastic moduli for the three samples are roughly equal, in the range of 20 MPa for splay-aligned samples. This can be deemed surprising considering that 4,4′-dithiodianiline should act as a dynamic crosslinker but is probably explained by its relatively strong absorption at the polymerization wavelength (365 nm), which may affect the photopolymerization process. However, we determined the gel fraction of LCE_30_ to be >80%, indicating successful photopolymerization even in the presence of high disulfide content (Fig. S4, ESI†). Based on the mechanical characterization, both the tensile strength and the fracture strain decrease upon addition of 4,4′-dithiodianiline, but less so for LCE_30_, possibly due to higher amount of unreacted amines within the polymerized network.^57^ However, thermal treatment of LCE_30_ at 100 °C for 3 days did not significantly change its mechanical properties (moduli of 20 and 22 MPa for fresh and heat-treated LCE_30_, respectively), indicating successful photopolymerization (Fig. S5, ESI†). Hence, we chose LCE_30_, combining higher fracture strain and higher 4,4′-dithiodianiline content, for further studies. The stress–strain curves for uniaxially aligned LCE_30_ are highly anisotropic (Fig. S6a and Table S1, ESI†), displaying linear deformation parallel to the director and slight soft elasticity and eventual strain-hardening perpendicular to the director.^18^ Anisotropy over large areas is confirmed by the polarized optical micrographs (Fig. S6b, ESI†). The power of the aza–Michael addition-based chain-extended LCE lies on the effective combination of control over LC alignment (planar, twisted, splay) and relatively high elasticity. This is in contrast to both glassy LCNs^60^ with superior alignment control but low fracture strain, and the two-step synthesis method or thiol–Michael chain extension that often require stretching of the sample to induce (planar) orientation.^61–63^

### Actuation performance

**Fig. 2:**
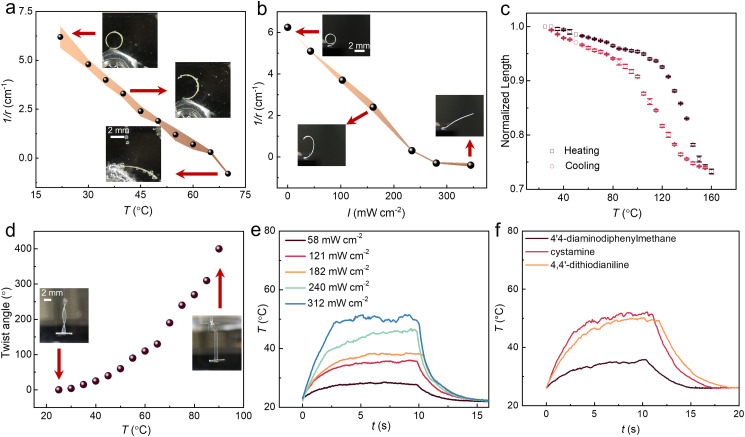
Actuation performance. (a) Bending of splay-aligned LCE_30_ strip at different temperatures and corresponding photographs (insets). *r*, radius of the bending arc. (b) Curvature change of a splay-aligned LCE_30_ strip as a function of irradiation intensity (385 nm) and corresponding photographs (insets). (c) Thermally induced contraction of planar-aligned LCE_30_ during one heating–cooling cycle. (d) Thermally induced twisting of twist-aligned LCE_30_ and corresponding photographs (insets). (e) Light-induced heating kinetics of LCE_30_ upon illumination with different intensities (385 nm). (f) Light-induced heating kinetics (385 nm, 240 mW cm^−2^) of planar-aligned LCEs with 4,4′-dithiodianiline, cystamine, and 4,4′-diaminodiphenylmethane (30 mol-% with respect to RM82) used as chain extenders/crosslinkers. Strip sizes in all experiments: 12 × 2 × 0.1 mm^3^.

As expected, the disulfide-containing LCEs respond to both heat and light (Fig. 2a and b). As the LCEs were polymerized at 63 °C, the splay-aligned strips spontaneously bent towards the homeotropic side at room temperature, and subsequent heating or UV irradiation (385 nm) resulted in unbending and a decrease in the radius of curvature. Fig. 2c shows the contraction of planar-aligned LCE_30_ strip as a function of temperature and corresponding expansion upon cooling, indicating an actuation strain of 25% at 160 °C. The order parameter of the sample was deduced by adding a small amount of dichroic dye, disperse red 1, into the polymerizable mixture before LCE fabrication, yielding a value of 0.51 for a planar-aligned sample (see Fig. S7, ESI,† for polarized absorption spectra). Fig. 2d illustrates the heat-driven twisting of a twist-aligned sample, yielding a twist angle of 400° upon heating to 90 °C. Fig. S8 (ESI†) presents the relationship between UV intensity and maximum temperature increase for splay-aligned LCE_30_. Corresponding heating kinetics is shown in Fig. 2e. As expected, the surface temperature increases and the deformation is accelerated upon increasing UV intensity. To confirm the central role of disulfide groups in the photothermal heating, we replaced the 4,4′-dithiodianiline with cystamine and 4,4′-diaminodiphenylmethane (Fig. S1, ESI†), while keeping their content in the LCE fixed. As shown in Fig. 2f, the photothermal temperature increase (385 nm, 240 mW cm^−2^) is similar for the 4,4′-dithiodianiline- and cystamine-containing LCEs and significantly reduced when using 4,4′-diaminodiphenylmethane. This highlights the dual role of the disulfide-containing chain extenders as both photothermal agents and dynamic crosslinkers. The photothermal actuation shows excellent reversibility, as no observable differences are detected after 120 light-driven actuation cycles (Fig. S9, ESI†).

### Welding experiments

**Fig. 3:**
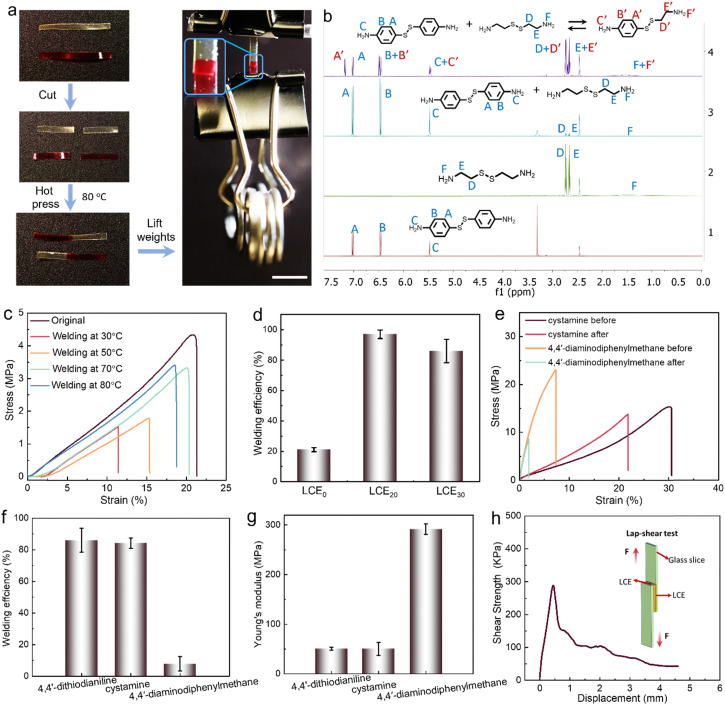
Welding of the disulfide-containing LCEs. (a) Photographs of welding of LCE_30_. After welding, the sample can withstand a 25 g weight without rupture. One LCE is stained by thermally diffusing disperse red 1 into the LCE for better visualization. (b) ^1^H NMR spectra of (1) 4,4′-dithiodianiline, (2) cystamine, (3) a mixture of 4,4′-dithiodianiline and cystamine, and (4) a mixture of 4,4′-dithiodianiline and cystamine after heating to 80 °C for 2 h in DMSO-d6. (c) Tensile stress–strain curves of splay-aligned LCE_30_ upon welding at different temperatures. (d) Welding efficiency of LCE_0_, LCE_20_, and LCE_30_ (welding conditions: 80 °C, 5 min). (e) Tensile stress–strain curves of LCEs with cystamine and 4,4′-diaminodiphenylmethane crosslinkers. (f) Welding efficiency and (g) Young's moduli of planar-aligned the 4,4′-dithiodianiline-, cystamine-, and 4,4′-diaminodiphenylmethane-containing LCEs, by stretching along the director axis. (h) Adhesion lap–shear testing for LCE_30_, where the shear strength is plotted against the displacement. Inset: The measurement setup.

As demonstrated in Fig. 3a, cut films of LCE_30_ can be welded together by hot pressing for 5 min at 80 °C. After welding, the sample can withstand a weight of 25 g, which is about 2500 times its own weight. The welding properties arise from the disulfide metathesis at elevated temperature. We confirmed this by comparing ^1^H NMR spectra of 4,4′-dithiodianiline, cystamine, and a mixture of 4,4′-dithiodianiline and cystamine before and after heating to 80 °C for 2 h in DMSO-d6.^60^ The new peaks appearing at 7.2 ppm (A′′), 6.5 ppm (B′′) and 5.5 ppm (C′′) for the mixture do not belong to 4,4′-dithiodianiline or cystamine but to the new compound 4-((2-aminoethyl)disulfaneyl)aniline formed upon bond exchange (Fig. 3b and Fig. S10–S13, ESI†) after 2 h at 80 °C, indicating the metathesis reaction of the disulfide bond (Fig. 3b).

Fig. 3c and Fig. S14a–g (ESI†) illustrate the representative stress–strain curves and welding efficiency after hot pressing at different temperatures, duration and contact area. The results show that the mechanical strength of the welded sample improves with increasing temperature and contact area. To prove that the disulfide metathesis reaction is responsible for the welding, we performed a swelling test (Fig. S14h, ESI†) and found that while the welded LCE_30_ quickly curled in acetone, after drying in air for 10 min it re-extended and remained undamaged, indicating strong chemical connection between the welded pieces. Fig. 3d shows the welding efficiency (as defined in the Experimental section) of LCE_0_, LCE_20_, and LCE_30_. Due to the lack of disulfide bonds in LCE_0_, the efficiency is only 20%, caused presumably by physical adhesion and slight sticking (Fig. 3d and Fig. S14f, ESI†). For LCE_20_ and LCE_30_, the corresponding efficiency is 95% and 85%, respectively, confirming the importance of the disulfide bonds in the welding process. By again replacing 4,4′-dithiodianiline with cystamine or 4,4′-diaminodiphenylmethane, we found that cystamine does not compromise the welding efficiency while no welding takes place in the 4,4′-diaminodiphenylmethane-containing LCE (Fig. 3e). This can be partially attributed to the higher modulus (50 MPa for 4,4′-dithiodianiline- and cystamine-containing LCEs, 290 MPa for 4,4′-diaminodiphenylmethane-containing LCE; Fig. 3f and Table S1, ESI†) and *T*_g_ (*ca.* 0 °C for 4,4′-dithiodianiline- and cystamine-containing LCEs, and 18 °C for 4,4′-diaminodiphenylmethane-containing LCE; Fig. S14i, ESI†) of the latter, which tend to negatively affect self-healing properties of elastomers.^61^ Furthermore, comparing the welding efficiency of LCE_30_ directly after welding and after 7 days or aging, no significant change in welding efficiency of samples was observed (Fig. S15, ESI†). Most of all, however, these results attest the necessity for the dynamic disulfide bonds in efficient welding. Finally, we performed adhesion lap–shear testing and peeling force per adhesive testing for LCE_30_, to demonstrate strong mechanical connection between the welded pieces. The lap–shear testing comprises two glass slides with LCE_30_ as middle adhesive (Fig. 3h). The shear strength of 0.3 MPa and shear modulus of 0.6 MPa confirm strong mechanical attachment due to successful welding (Fig. 3h and Fig. S14g, ESI†).

### Shape morphing after welding

**Fig. 4:**
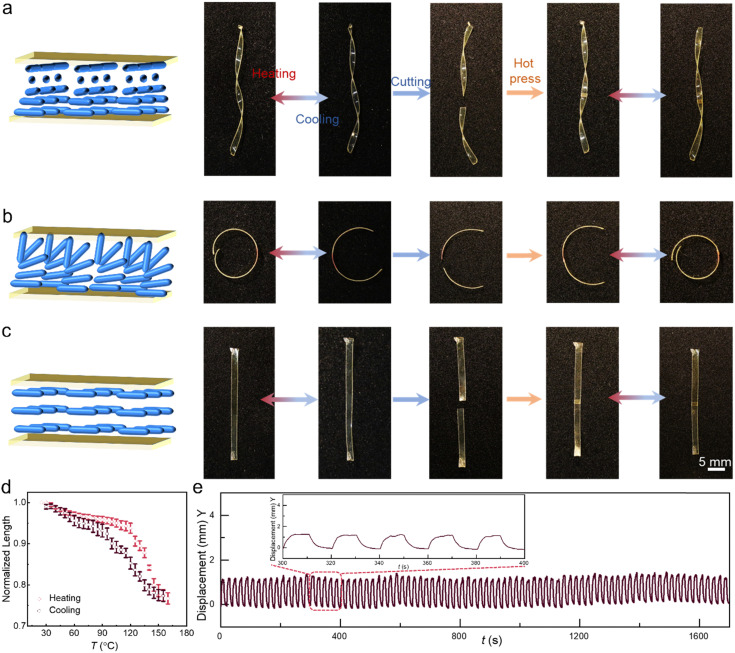
Shape morphing after welding. Schematic diagram of (a) twisted, (b) splayed, and (c) planar alignment and photographs of actuation before and after cutting, pasting, and welding at 80 °C for 5 min. (d) Thermal contraction of welded, planar-aligned LCE_30_ during one heating–cooling cycle. (e) The deformation reversibility of a bending LCE_30_ strip after welding upon cyclic light excitation. Light: 385 nm, 120 mW cm^−2^.

**Fig. 5:**
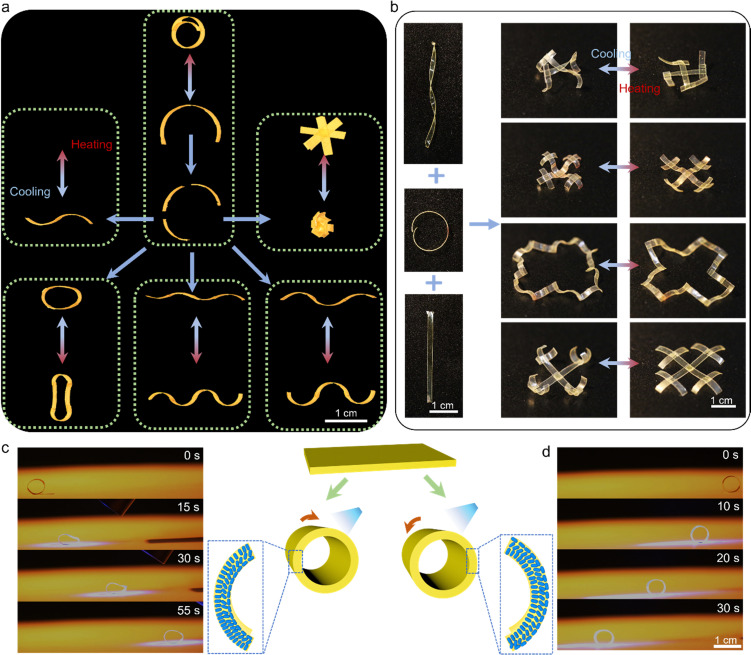
Versatile shape transformations in welded LCE strips. (a) Examples of different actuator structures prepared by welding, including “S”-shape, wavy, round, and petal shapes and their heat-induced deformations. (b) Complex actuator assemblies by welding splayed, twisted, and planar-aligned LCE strips together. Snapshots of a strip ring rolling towards (c) and away from (d) the light source. Schematic illustration and photograph of a ring obtained through welding the two ends of a splay-aligned LCE strip (middle). The LCE is made by fixing its heat-induced bending direction towards the inner (c) or outer (d) surface. Irradiation conditions: 385 nm, 160 mW cm^−2^.

To demonstrate the utility of our concept in LCE actuation control, we welded differently aligned (planar, splay, twisted) LCE pieces together (Fig. 4a–c). The surface-aligned samples were cut into two pieces and hot-pressed at 80 °C for 5 min, after which their actuation performance was characterized. A planar sample was chosen for heat-induced deformation to test the influence of welding on the material deformability. Fig. 4d shows a contraction strain of about 23% at 150 °C, which is similar to the pre-welded sample (Fig. 2c; 25% at 150 °C). A splay-aligned sample was chosen for cyclic actuation tests, evincing no fatigue at least over 80 actuation cycles even after welding (Fig. 4e). The reversible actuation of welded, differently aligned LCEs is attributed to the fact that the LC alignment is fixed by the photopolymerization and not destroyed during the welding process, so no post-treatment is required to regain the alignment again after welding. Cross-sectional POM images of the planar LCE_30_ after welding confirm that the alignment is maintained after welding (Fig. S16, ESI†). This is an important difference to many previous reports, which require re-stretching to retain the LC orientation,^42,43,45,48,52^ a process which becomes redundant in surface-aligned LCEs. This allows us to, *e.g.*, cut splay-aligned LCE into segments and weld them together to form various 3D constructs that can be thermally actuated (Fig. 5a). Furthermore, as shown in Fig. 5b and Fig. S17 (ESI†), splayed, twisted, and planar segments can be assembled into diverse configurations to obtain complex shape-morphing. And since the 4,4′-dithiodianiline absorbs UV light, these actuation modes can be achieved remotely in response to light, *via* photothermal heating (Fig. S18 and Movie S1, ESI†). The welding strategy also enables constructing splay-aligned LCE “wheels” exhibiting either positive or negative phototaxis under UV illumination (Fig. 5c, d and Movies S2, S3, ESI†), depending on whether the two ends of the strip are welded homeotropic side inwards or outwards. Hence, self-sustained rolling motion is obtained without the use of glue or bilayer fabrication strategies.^48,64^

## Conclusions

We demonstrate dynamic chain-extended liquid crystal elastomers based on aza–Michael addition reaction that can be arbitrarily welded while maintaining their molecular alignment (planar, twisted, splay) as dictated by surface alignment. This can be attributed to dynamic disulfide exchange provided by 4,4′-dithiodianiline crosslinks, confirmed by NMR studies and by control experiments where 4,4′-dithiodianiline is replaced with 4,4′-diaminodiphenylmethane. The design enables welding surface-aligned LCE segments into complex actuating structures, where the disulfide-containing crosslinks also act as photothermal absorbers that enable light-driven actuation of the LCE constructs. The results provide a facile method to fabricate arbitrarily aligned, complex-shaped LCE actuators with efficient welding properties, offering new alternatives for the development of future smart materials and LCE-based soft robots.

## Conflicts of interest

There are no conflicts to declare.

## Supplementary Material

TC-013-D5TC01194A-s001

TC-013-D5TC01194A-s002

TC-013-D5TC01194A-s003

TC-013-D5TC01194A-s004

## Data Availability

The data supporting this article have been included as part of the ESI.† The raw data will be available upon request from the authors.
