# Supplementary material for: Welding complex-shaped actuators from dynamic liquid crystal elastomers
Source: J Mater Chem C Mater. 2025 May 21;13(26):13482–9. doi: 10.1039/d5tc01194a (PMC12135048; doi:10.1039/d5tc01194a)
Supplement: TC-013-D5TC01194A-s001 [file TC-013-D5TC01194A-s001.pdf]

Supplementary Information (SI) for Journal of Materials Chemistry C.
This journal is © The Royal Society of Chemistry 2025

Supporting Information

Welding complex-shaped actuators from dynamic liquid crystal elastomers

Jie Jiang, Hongshuang Guo, Hao Zeng, Arri Priimagi**

Dr. J. Jiang, Dr. H. Guo, Dr. H. Zeng and Prof. A. Priimagi

Smart Photonic Materials, Faculty of Engineering and Natural Sciences, Tampere University, P.O. Box 541, FI-33101 Tampere, Finland.

*Correspondence to: hongshuang.guo@tuni.fi, arri.priimagi@tuni.fi

Keywords: Dynamic covalent chemistry; Disulfide bonds; Liquid crystal elastomer; Welding; Complex actuation; Light responsive

Contents:

1. Experimental section and method
2. Supplementary figures S1 to S18.
3. Captions for movies

1. Experimental section and method

Materials: 1,4-Bis-[4-(6-acryloyloxyhexyloxy)benzoyloxy]-2 methylbenzene (RM82, 99%) was purchased from SYNTHON Chemicals GmbH & Co. 4,4'-dithiodianiline, dodecylamine, phenylbis(2,4,6-trimethylbenzoyl)phosphine oxide (I-819) and cystamine dihydrochloride were purchased from TCI. 4,4'-diaminodiphenylmethane was purchased from Sigma Aldrich. All chemicals were used as received.

LCE film preparation: For planar-aligned samples two rubbed PVA-coated (poly(vinyl alcohol); 5% water solution, spin coating at 4000 RPM for 1 min, baking at 100 ºC for 10 min) glass slides with parallel rubbing directions are glued together. For twisted samples the procedure is the same, but the rubbing directions are perpendicular. For splayed samples one glass slide was coated with homeotropic alignment layer (JSR OPTMER, 4000 RPM for 1 min, followed by baking at 100 ºC for 10 min and 180 ºC for 30 min) and a rubbed glass slide were glued together. Spacers: 100 μm microspheres (Thermo scientific).

Liquid crystal mixtures were prepared by mixing 0.1 mmol RM82, 0-0.08 mmol dodecylamine, 0-0.08 mmol 4,4'-dithiodianiline, cystamine or 4,4'-diaminodiphenylmethane, and 2.5 wt% of I-819 at 90 ºC. The mixtures were filled into the LC cells via capillary effect at 90 ºC and cooled down to 63 ºC (1 ºC min^{-1}). The cells were put in an oven to allow the Aza–Michael addition reaction for oligomerization for 24 h at 63 ºC, and polymerized under UV (365 nm, 180 mW/cm^2, 30 min). Finally, the cells were opened with a blade and strips with desired dimensions were cut from the film.

Material Characterization: ^1H NMR spectra were recorded on a 500 MHz (JEOL ECZR 500) instrument at 25 ºC using dimethyl sulfoxide-d6 (DMSO-d6, Sigma-Aldrich) as the solvent. The Fourier Transform Infrared (FTIR) spectra were recorded on a Perkin Elmer Spectrum Two Fourier Transform Infrared between 4000 and 650 cm^{-1} with a clean ATR crystal used as a reference. UV-Vis absorption spectra were recorded with an Agilent Cary 60 spectrophotometer. Differential Scanning Calorimetry (DSC) measurements were performed with a Netzsch DSC 214 Polyma instrument at a heating/cooling rate of 10 ºC/min. The measurements were performed with 6-10 mg of sample at 1 bar under nitrogen atmosphere (flow rate of 20 mL/min) in the temperature range between -50 ºC and 150 ºC. Stress−strain curves were determined with a homemade tensile tester in a 0.1 mm thick film with a stretching speed of 0.05 mm/s.

Optical characterization: Optical images and relevant research videos were recorded by using a Canon 5D Mark III camera with a 100 mm lens. Thermal images were recorded with an infrared camera (FLIR T420BX) equipped with a close-up (2×) lens. The LED light source used is CoolLED pE-4000. The related data were analyzed with video analysis software (Kinovea).

Welding test: To visualize the welding effect, we placed one of the films on a 100 ºC hot plate, and then diffused Disperse Red 1 dye into the LCE to change its color. The LCE was then cut into two pieces that were hot-pressed at different temperatures and for different durations, before conducting mechanical and actuation performance testing. The welding efficiency (%) was defined as the proportion of restored toughness to the original toughness on account of the recovery of both stress and strain. All experiments were repeated at least three times using the same sample to obtain statistically reliable data.

Preparation of complex shapes: Cut planar, twisted, and splayed samples of different lengths were prepared. The samples with different LC alignments were welded together (80 ºC, 5 min) to obtain various two-and three-dimensional shapes (Figure S17) that were further (photo)actuated.

2. Supplementary figures

a

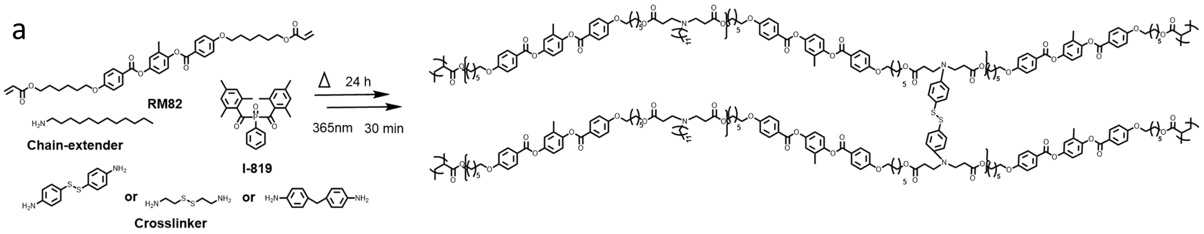

b

	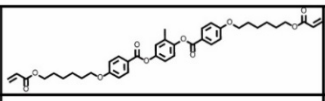	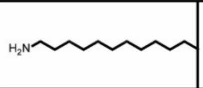	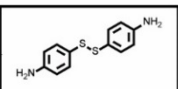	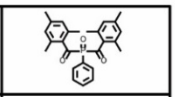
LCE_0	1	0.8	0	2.5wt%
LCE_{20}	1	0.4	0.2	2.5wt%
LCE_{30}	1	0.2	0.3	2.5wt%

Figure S1. (a) Synthetic route for the chain-extended LCE with different crosslinkers (from left to right: 4,4'-dithiodianiline, cystamine, 4,4'-diaminodiphenylmethane). (b) The chemical composition for the LCE samples studied.

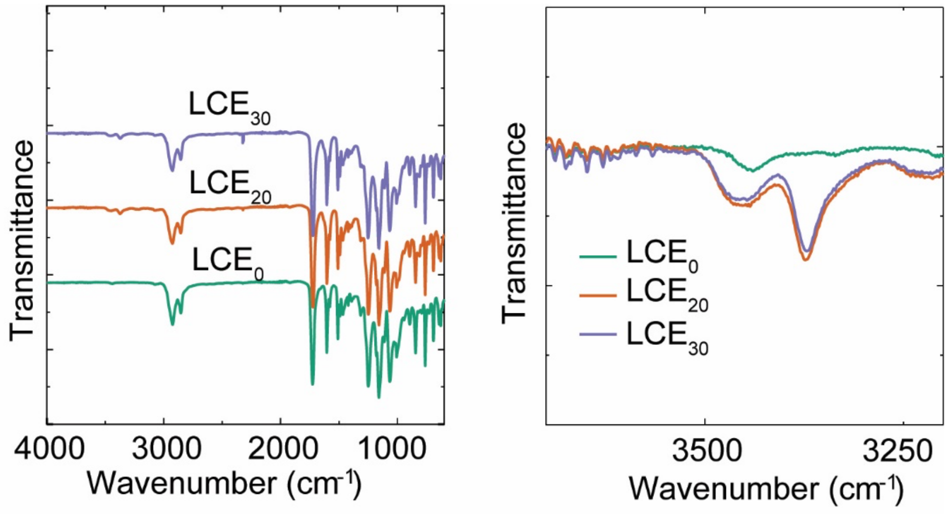

Figure S2. FTIR spectra of LCE_0, LCE_{20}, and LCE_{30}.

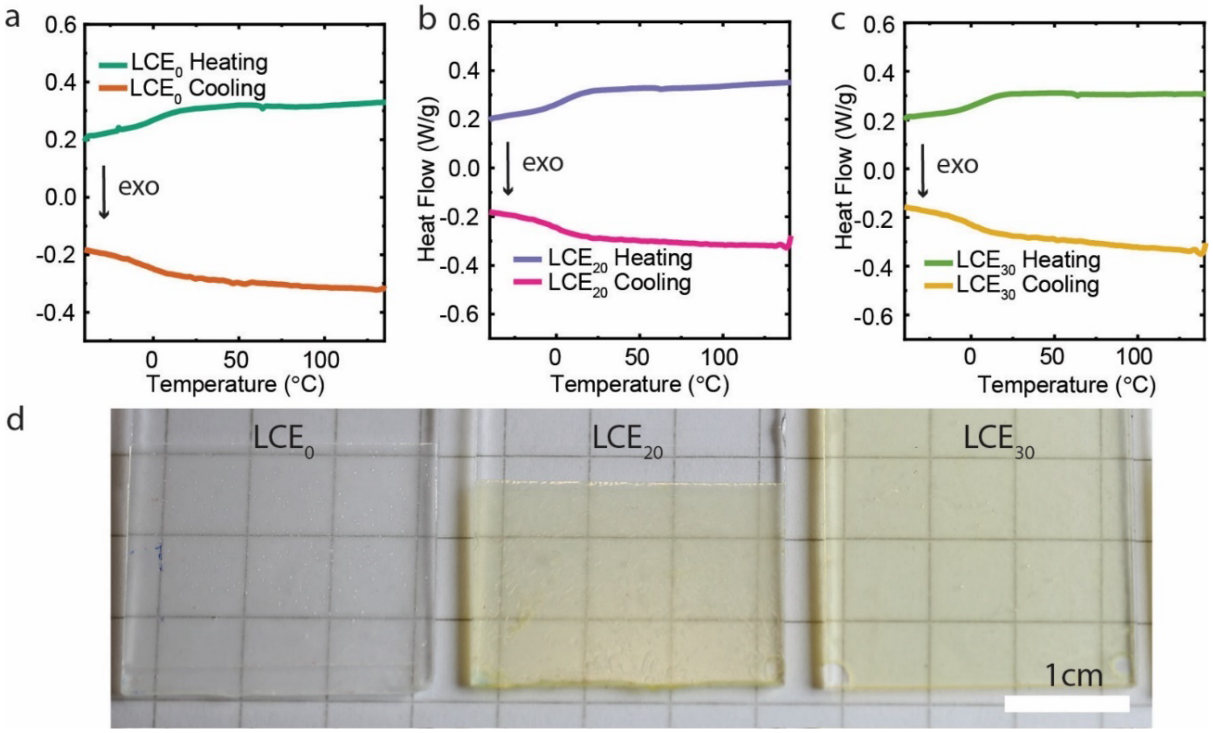

Figure S3. DCS curves of LCE$_0$, LCE$_{20}$, and LCE$_{30}$ during the second heating and cooling cycles (10 °C min^{-1}). (a) LCE$_0$, (b) LCE$_{20}$, (c) LCE$_{30}$. (d) Photographs of LCE$_0$, LCE$_{20}$, and LCE$_{30}$.

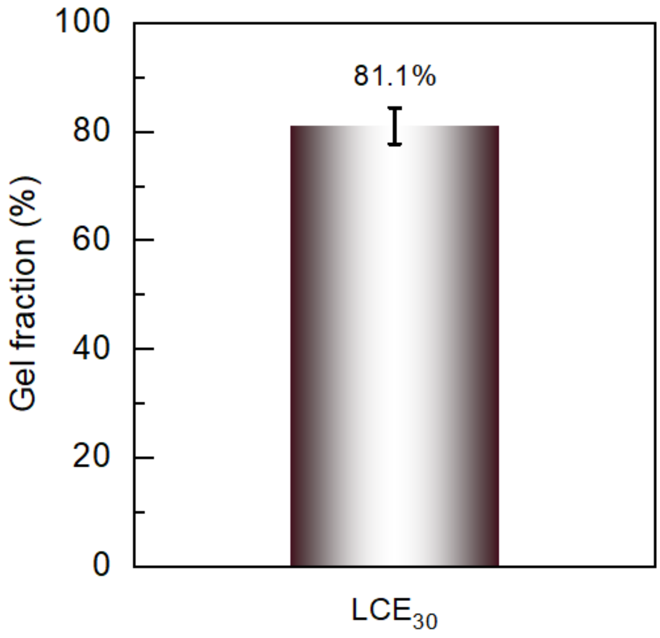

Figure S4. Gel fraction of LCE$_{30}$. To measure the gel fraction, the sample was placed in a vial containning 3 mL of toluene and left at room temperature for 24 h. The sample was dried at 100 ºC until and its weight was ompared to the original sample weight. The gel fraction was calculated as $W_{dry}/W_{original} \times 100$, where $W_{original}$ represents the initial weight of the sample, while W_{dry} represnts the weight of the dried, insoluble portion of the sample.

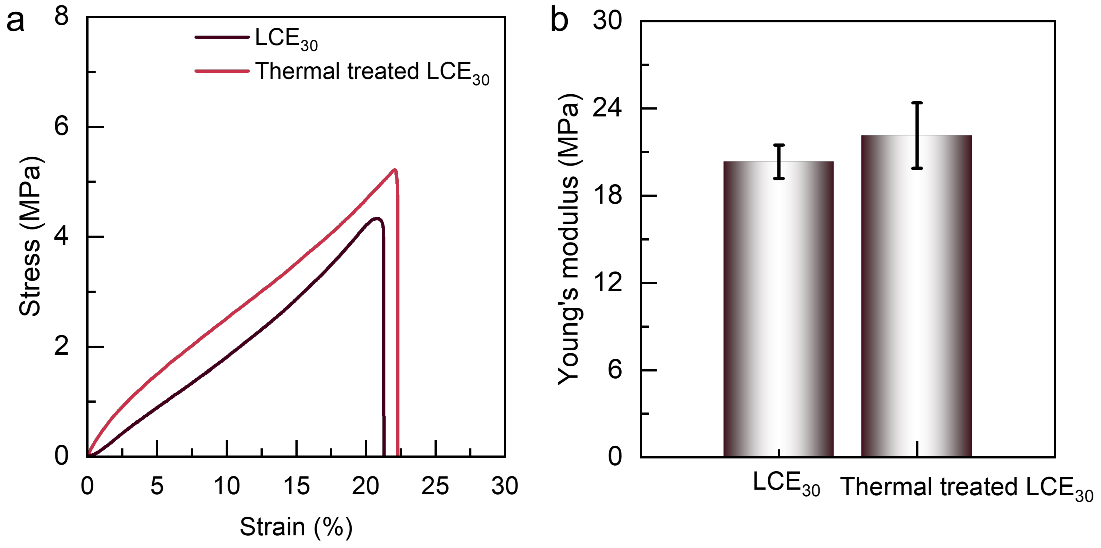

Figure S5. (a) Stress-strain curve and (b) Young's modulus curve of LCE30 after heating treatment at 100 °C for 3 days.

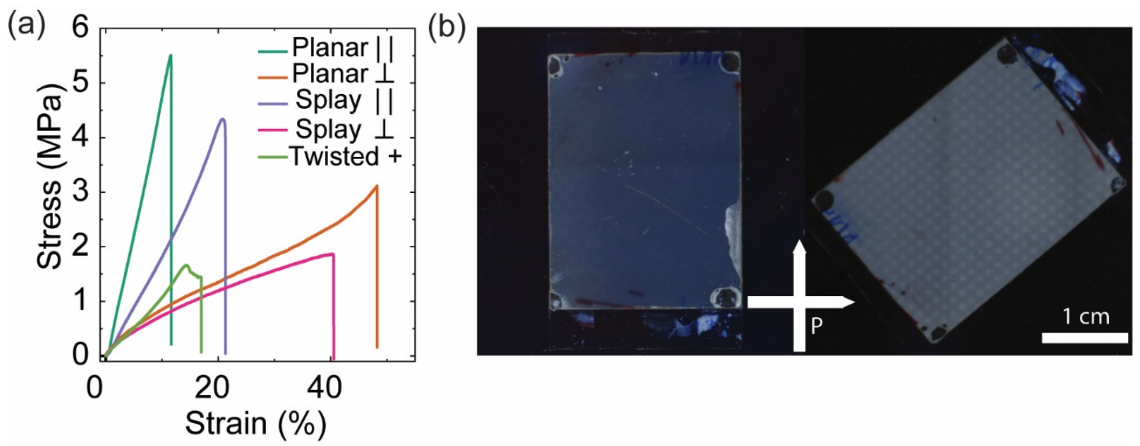

Figure S6. (a) Tensile stress–strain curves of LCE_{30} with different nematic director orientations. || – parallel to the director; ⊥ – perpendicular to the director; + –twisted alignment, with stretching direction set at 45° respect to the directors. (b) Cross-polarized images of splay-aligned LCE_{30}. The arrows indicate the polarizer and analyzer directions.

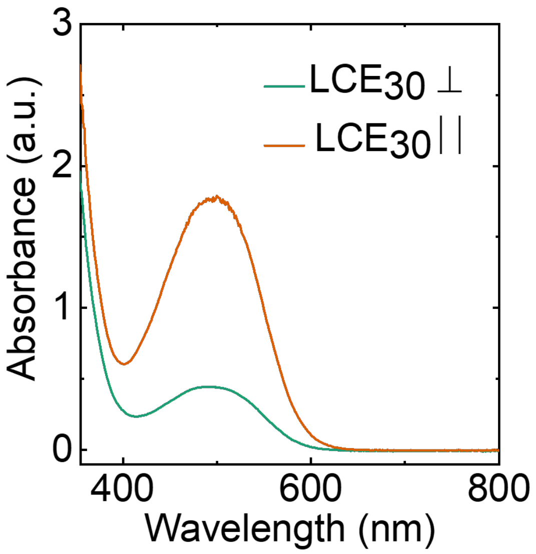

Figure S7. Polarized absorption spectra of planar-aligned LCE_{30} with 0.5 % DR1 added. || represents LC orientation parallel to the director and ⊥ in the perpendicular direction. The order parameter as $S_p = A|| - A\perp /A|| + 2A\perp$, where $A||$ and $A\perp$ are the measured absorbance values with light polarized parallel and perpendicular to the LC alignment, respectively. S_p is determined to be 0.51 for the homogeneously aligned sample, by averaging the data in wavelength range 475-525 nm.

Table S1. Anisotropic mechanical properties of LCE_{30}.

**Code**	**E_Y (MPa)**	**σ_{max} (MPa)**	**ε_{max} (%)**
Planar ||	50.7±3	5.2±0.2	10±1
Planar	8.9±0.7	2.5±0.6	40±7
Splay ||	17.5±2.2	4±0.4	20±1
Splay	6.4±0.4	1.6±0.3	34±6
Twisted +	8.4±1.2	1.4±0.2	17±1

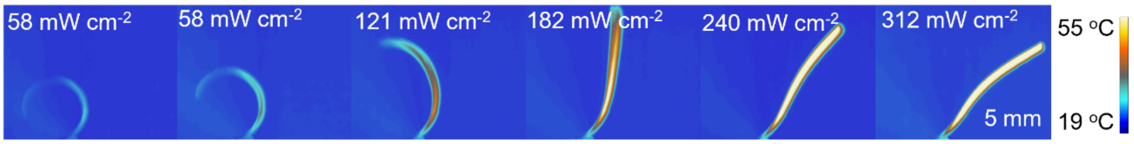

Figure S8. Thermal camera images of LCE_{30} film upon 385 nm illumination with different light intensity.

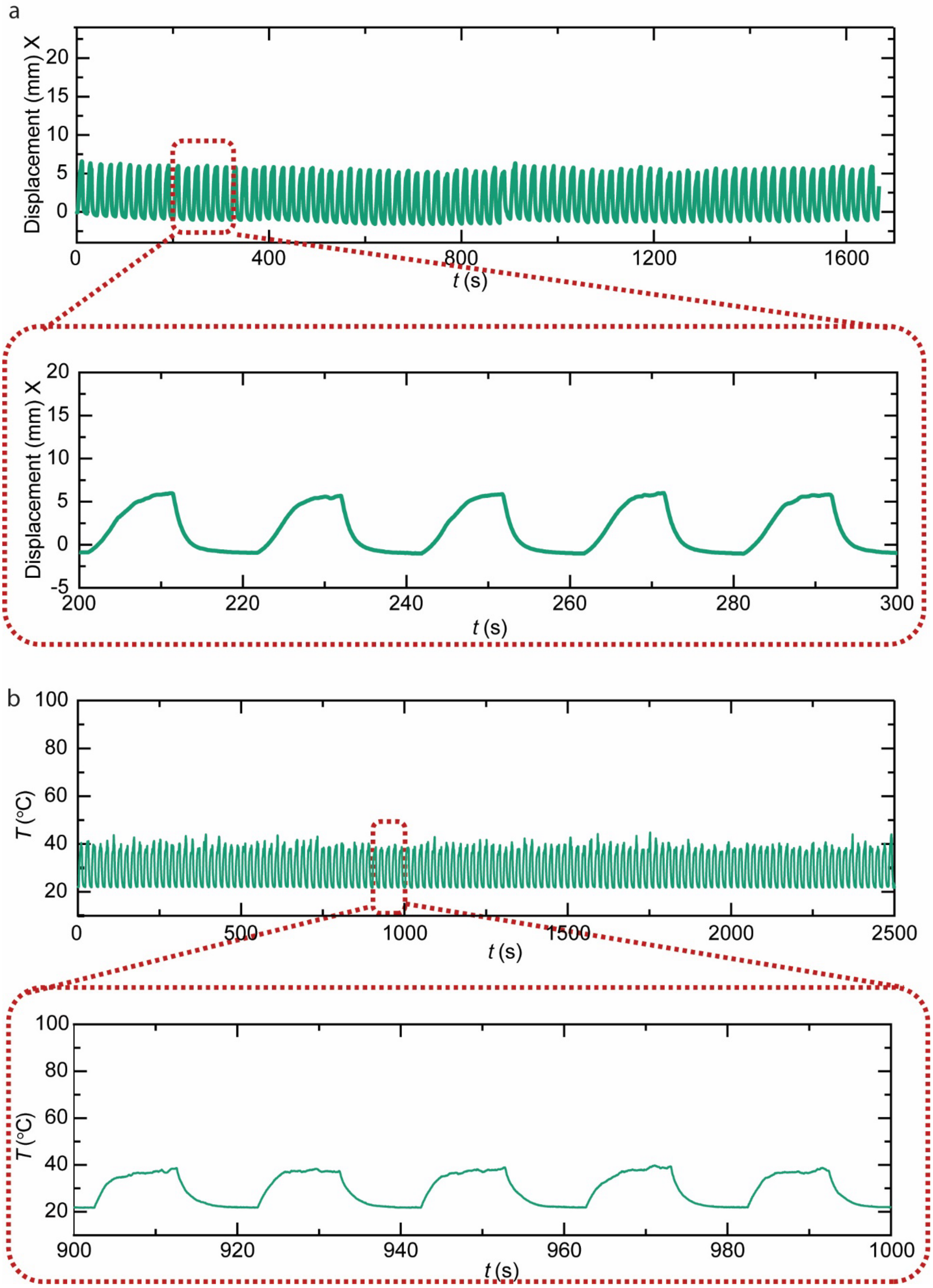

Figure S9. (a) Deformation reversibility of a bending strip upon cyclic light excitation. Light: 385 nm, 120 mW cm^{-2}. (b) The photothermal heating kinetics during cyclic light excitation. Light: 385 nm, 180 mW cm^{-2}.

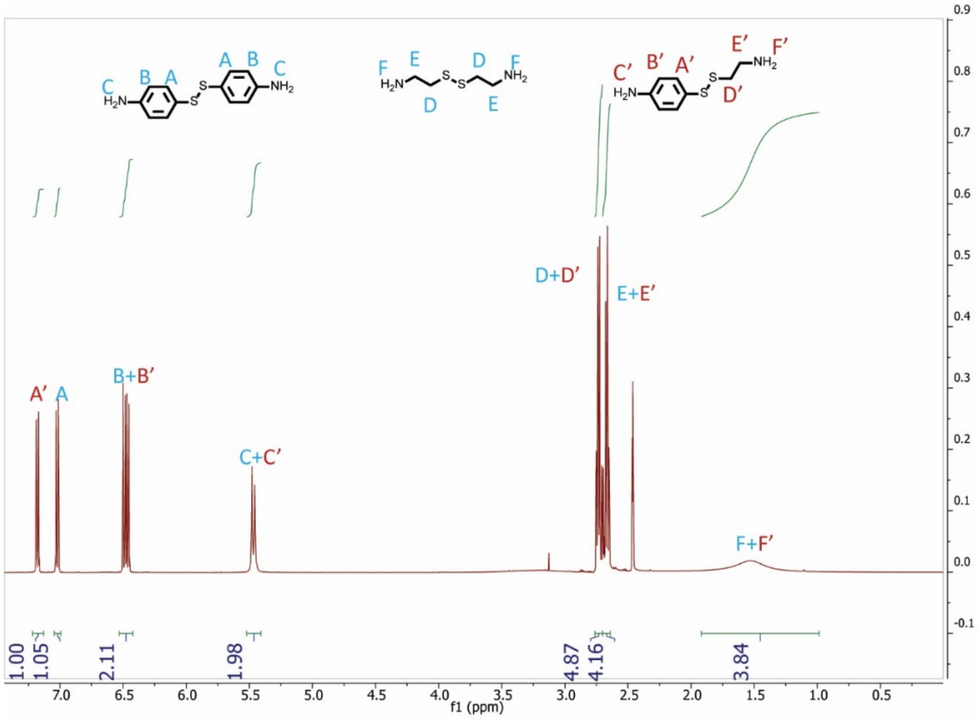

Figure S10. ^{1}H NMR spectra of mixture 4,4'-dithiodianiline and cystamine after heating at 80 °C for 2 h in DMSO-d6.

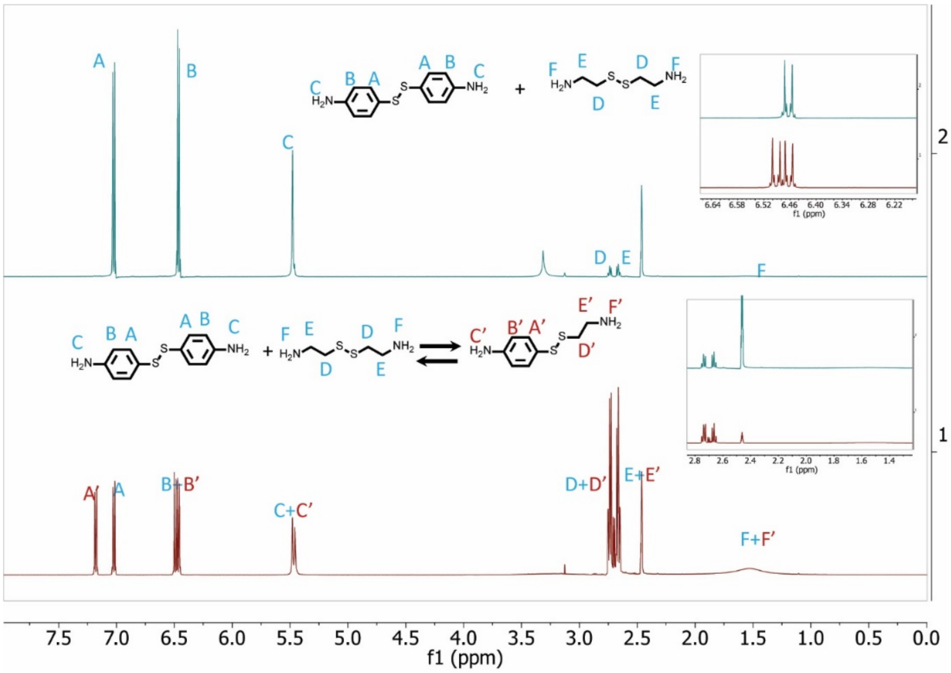

Figure S11. ^{1}H NMR spectra of mixture of 4,4'-dithiodianiline and cystamine before (top) and after (bottom) heating at 80 °C for 2 h in DMSO-d6.

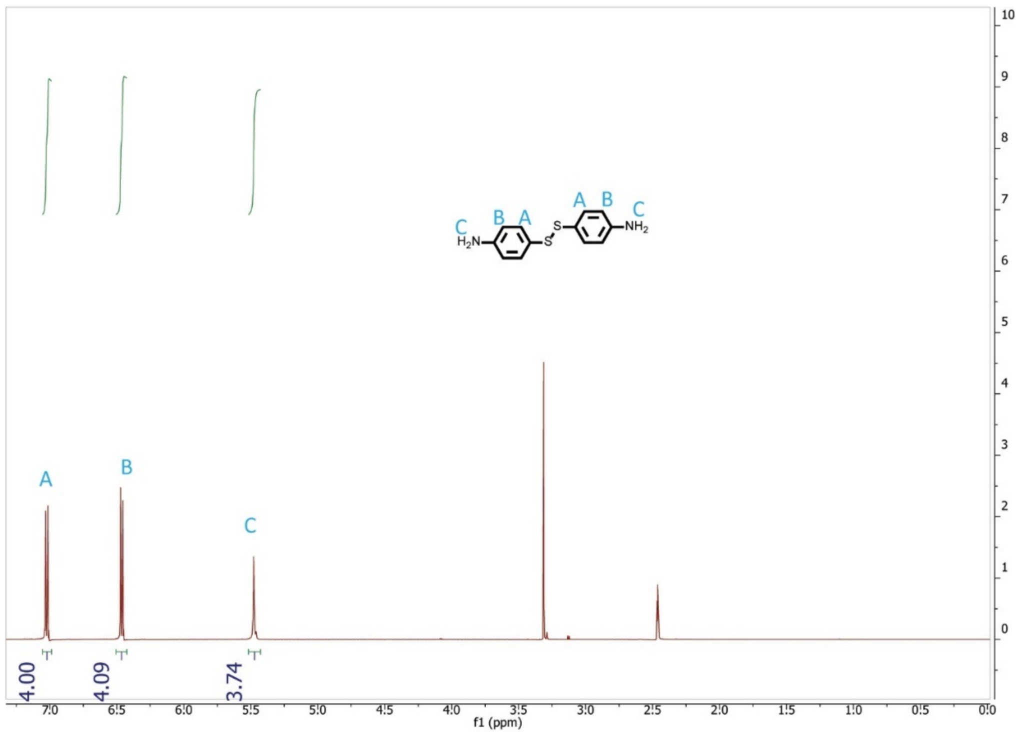

Figure S12. ^{1}H NMR spectra of 4,4'-dithiodianiline in DMSO-d6.

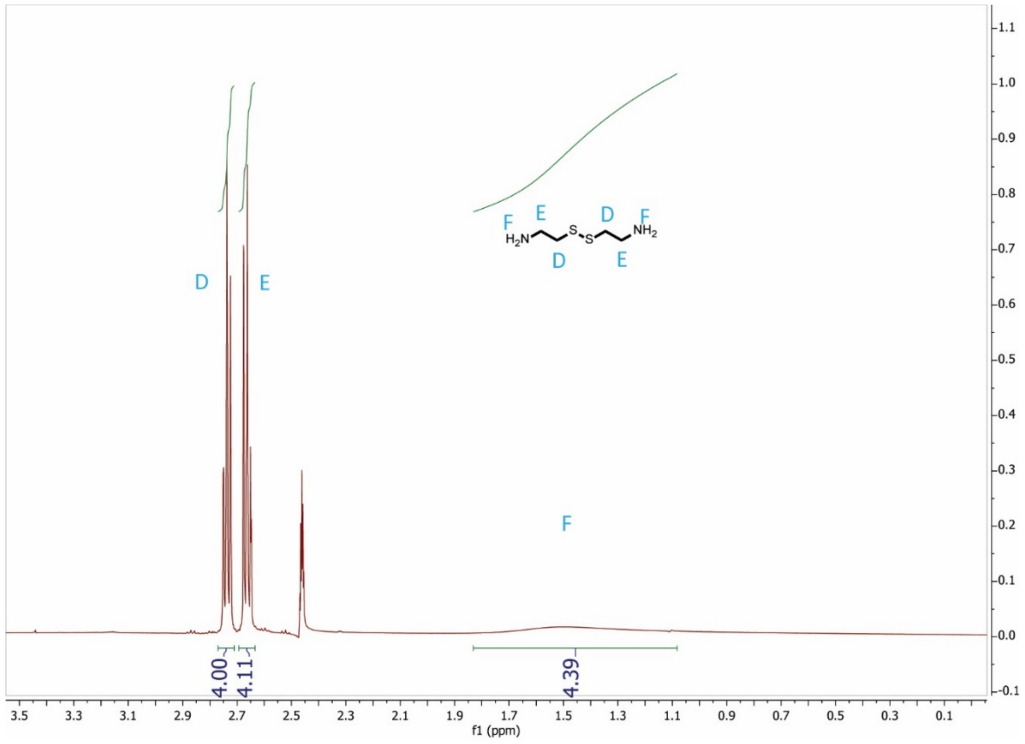

Figure S13. ^{1}H NMR spectra of cystamine in DMSO-d6.

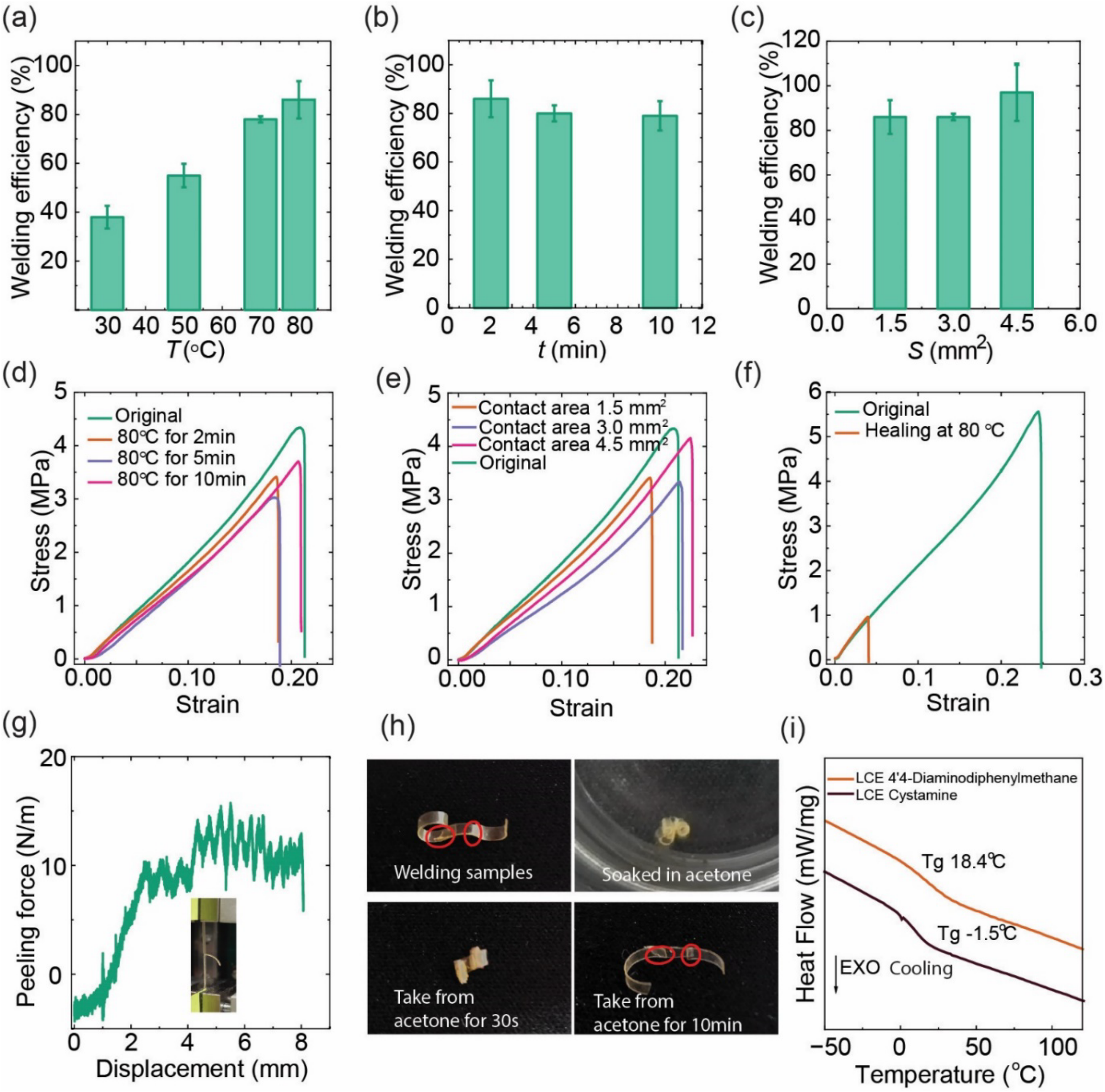

Figure S14. (a) Welding efficiency of LCE_{30} strips welded at different temperatures for 5 min. (b) Welding efficiency of LCE_{30} strips at 80 ºC for different times. (c) Welding efficiency of LCE_{30} strips under hot pressing with different contact areas. Tensile stress–strain curves of original and welded LCEs for (d) different welding durations, and (e) contact areas. (f) Stress–strain curves of LCE_0 before and after hot pressing at 80 ºC for 5 min. (g) Curve of double peeling force per adhesive width between the two LCE_{30} films (inset: photos of the peeling process). (h) Welding stability of LCE_{30} after soaking and swelling in acetone and subsequent drying. (i) DSC curves of LCEs containing 4,4'-diaminodiphenylmethane and cystamine at a heating/cooling rate of 10 ºC/min.

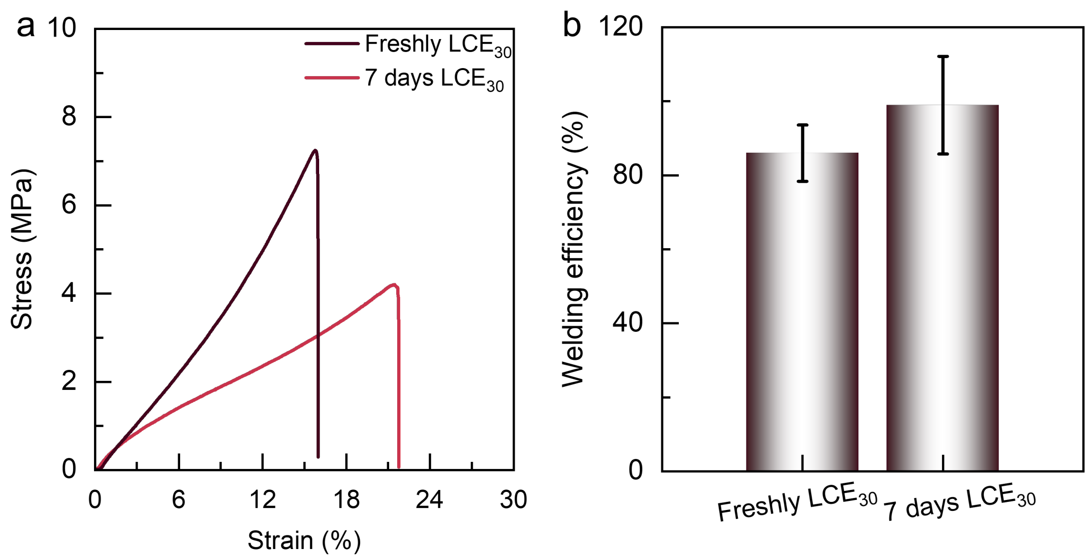

Figure S15. (a) Stress-strain curve and (b) Young's modulus curve of LCE_{30} after welding for 7 days.

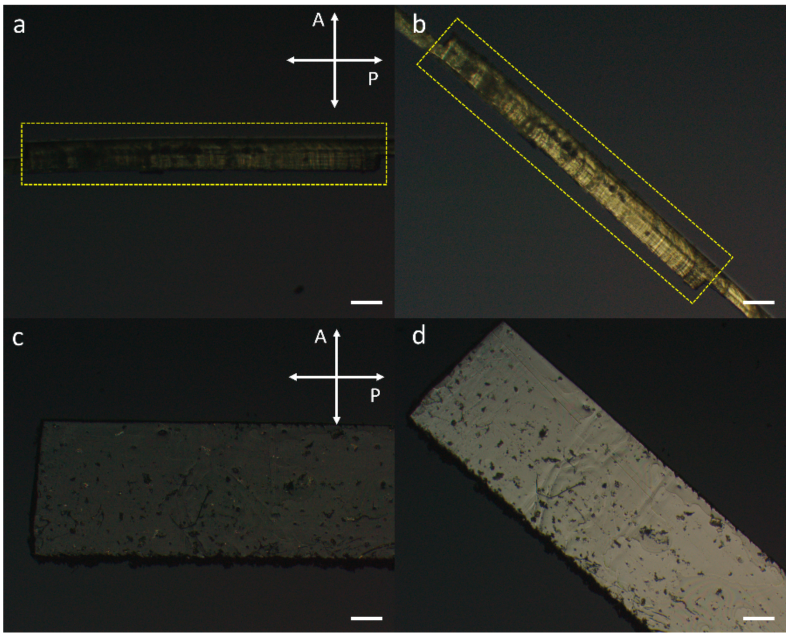

Figure S16. Cross-sectional POM images of the interface between two overlaped planar LCE films after welding at (a) 0° and (b) 45 ° angles between the director nad the polarizer axis. The yellow box marks the welded are. (c), (d) Corresponding POM images of the welded area from top view. Scale bar: 200 μm.

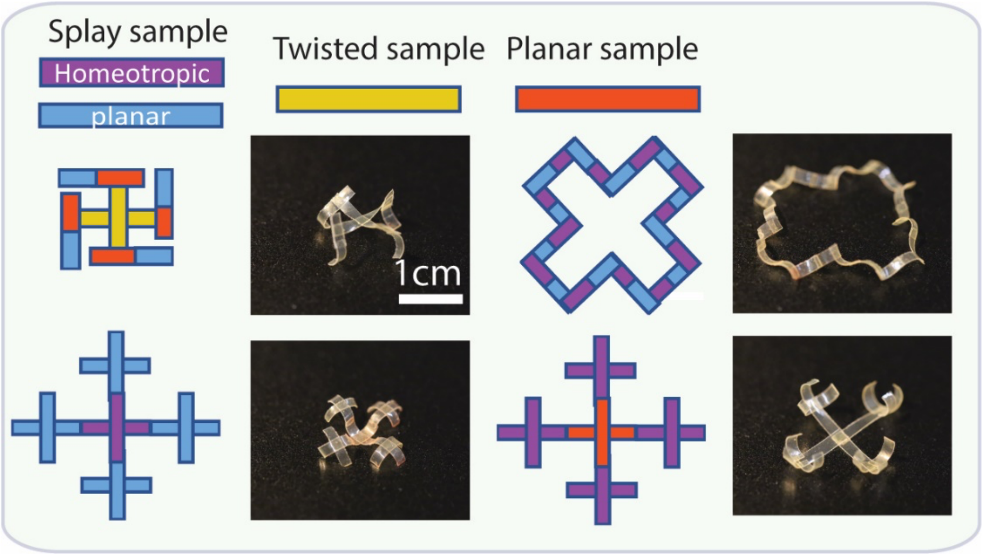

Figure S17. Design of complex LCE assemblies by welding splayed, twisted, and planar LCE strips.

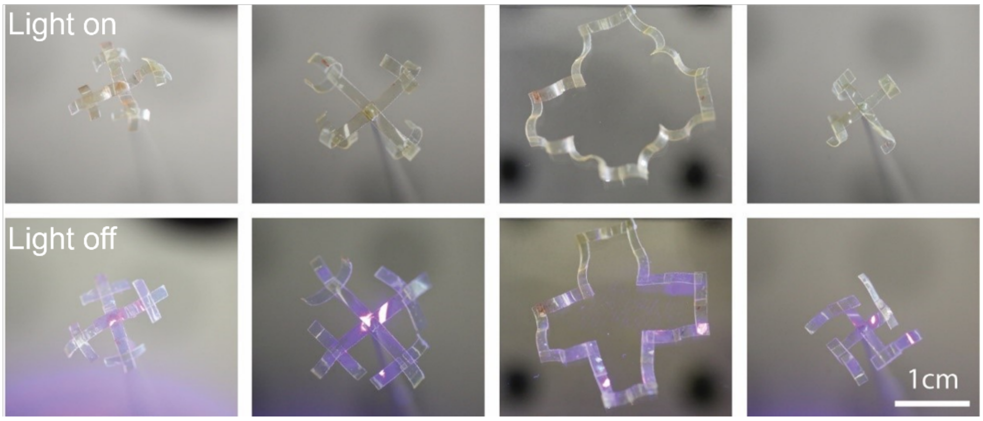

Figure S18. Complex LCE assemblies and their photothermally induced deformations (385 nm, 160 mW cm^{-2}).

3. Captions for supporting movies

Movie S1. Light-controlled shape-morphing in complex LCE assemblies. Light irradiation: 385 nm, 240 mW cm^{-2}.

Movie S2. Light-controlled LCE ring rolling toward the light. Light irradiation: 385 nm, 160 mW cm^{-2}. LCE strip dimensions: $20\times3\times0.1$ mm^3.

Movie S3. Light-controlled LCE ring rolling away from the light. Light irradiation: 385 nm, 160 mW cm^{-2}. LCE strip dimensions: $20\times3\times0.1$ mm^3.
